# Future climate stimulates population out-breaks by relaxing constraints on reproduction

**DOI:** 10.1038/srep33383

**Published:** 2016-09-14

**Authors:** Katherine A. Heldt, Sean D. Connell, Kathryn Anderson, Bayden D. Russell, Pablo Munguia

**Affiliations:** 1 Southern Seas Ecology Laboratories, School of Biological Sciences, The University of Adelaide, Adelaide 5005 SA, Australia; 2 Department of Zoology, The University of British Columbia, Vancouver, V6T 1Z4 BC, Canada; 3 The Swire Institute of Marine Science and School of Biological Sciences, The University of Hong Kong, Hong Kong SAR, China

## Abstract

When conditions are stressful, reproduction and population growth are reduced, but when favourable, reproduction and population size can boom. Theory suggests climate change is an increasingly stressful environment, predicting extinctions or decreased abundances. However, if favourable conditions align, such as an increase in resources or release from competition and predation, future climate can fuel population growth. Tests of such population growth models and the mechanisms by which they are enabled are rare. We tested whether intergenerational increases in population size might be facilitated by adjustments in reproductive success to favourable environmental conditions in a large-scale mesocosm experiment. Herbivorous amphipod populations responded to future climate by increasing 20 fold, suggesting that future climate might relax environmental constraints on fecundity. We then assessed whether future climate reduces variation in mating success, boosting population fecundity and size. The proportion of gravid females doubled, and variance in phenotypic variation of male secondary sexual characters (i.e. gnathopods) was significantly reduced. While future climate can enhance individual growth and survival, it may also reduce constraints on mechanisms of reproduction such that enhanced intra-generational productivity and reproductive success transfers to subsequent generations. Where both intra and intergenerational production is enhanced, population sizes might boom.

When environmental conditions are stressful, reproduction and population growth are delayed^1^, but when favourable, reproduction and population size can boom^2^. The effects of future climate on animal populations are often considered within the context of strong negative effects while strong positive effects are less considered. Ocean warming and acidification are considered stressors^3^,^4^ through increasing metabolic costs of individuals^5^,^6^,^7^. Nonetheless, elevated temperature can have positive effects through an increase in metabolism^8^ enabling population growth^9^ when elevated resources brought by carbon enrichment^10^ meet metabolic demands. It is possible that future climate may not only relax constraints on population growth, but also enable some populations to boom.

Herbivore populations appear particularly sensitive to future climate as meta-analyses suggest a general decrease in population size^11^,^12^. Yet, while unusual, there is evidence for population growth in herbivores^12^, which could be through the relaxation of abiotic and biotic constraints on reproductive output. Indeed, elevated CO_2_ and temperature can enhance food intake as a function of increasing per capita herbivory^13^,^14^. Such conditions may not only enhance reproductive success, but over successive generations they may also yield substantive increases in population sizes. While future conditions enhance foraging resources^13^ and survival amongst predators^15^, little is known about the contribution of reproductive success to population growth. Where reproductive success adjusts to favourable environmental conditions, the relaxation of competitive restriction to mates becomes a critical mechanism^16^,^17^,^18^.

Intensifying intraspecific interactions buffer runaway reproductive success as populations become increasingly dense^19^. Increasing competition for mates reduces male access to mates, and less competitive males are unable to reproduce^20^. Females play a particularly strong role in determining male mating success across a broad spectrum of taxa (e.g. territorial birds^21^, fish^22^, and crustaceans^23^). Females can increase reproductive output when mating with high quality males^24^ or reduce the intensity of male-male competition to increase overall reproductive success^16^.

As a test of this enhanced reproduction mechanism, we propose that the relaxation of abiotic constraints imposes strong directional selection on male ornaments which increase access to mates as male sexually selected traits become more exaggerated and homogenized^25^,^26^. Access to mates is gained when individuals deploy an array of successful mating strategies arising from sexual selection often producing dissimilar phenotypes^18^,^27^,^28^. Phenotypic traits are costly to maintain in stressful environments^29^,^30^,^31^, and males with the most appealing traits will monopolize females^18^,^28^. We consider, therefore, that as environmental constraints are relaxed, a higher proportion of males can maintain costly traits, predicting reduced variation in sexually selected characters^32^ and an increase reproductive success^33^ that together results in population growth^18^,^34^.

The development of theory on future climate as a facilitator of population growth lags behind that of climate as a stressor causing population decline. While the idea that future climate need not constrain population size is well known^12^, there are few tests of hypotheses that predict population growth, particularly the mechanisms enabling growth via enhanced reproductive potential. We tested whether elevated temperature and carbon dioxide could increase population growth of a herbivorous amphipod (*Cymadusa pemptos*), and to account for this population increase we observed reproductive potential by testing the prediction that phenotypic variation in male secondary sexual characters is reduced while females fecundity is enhanced.

## Results

**Figure 1:**
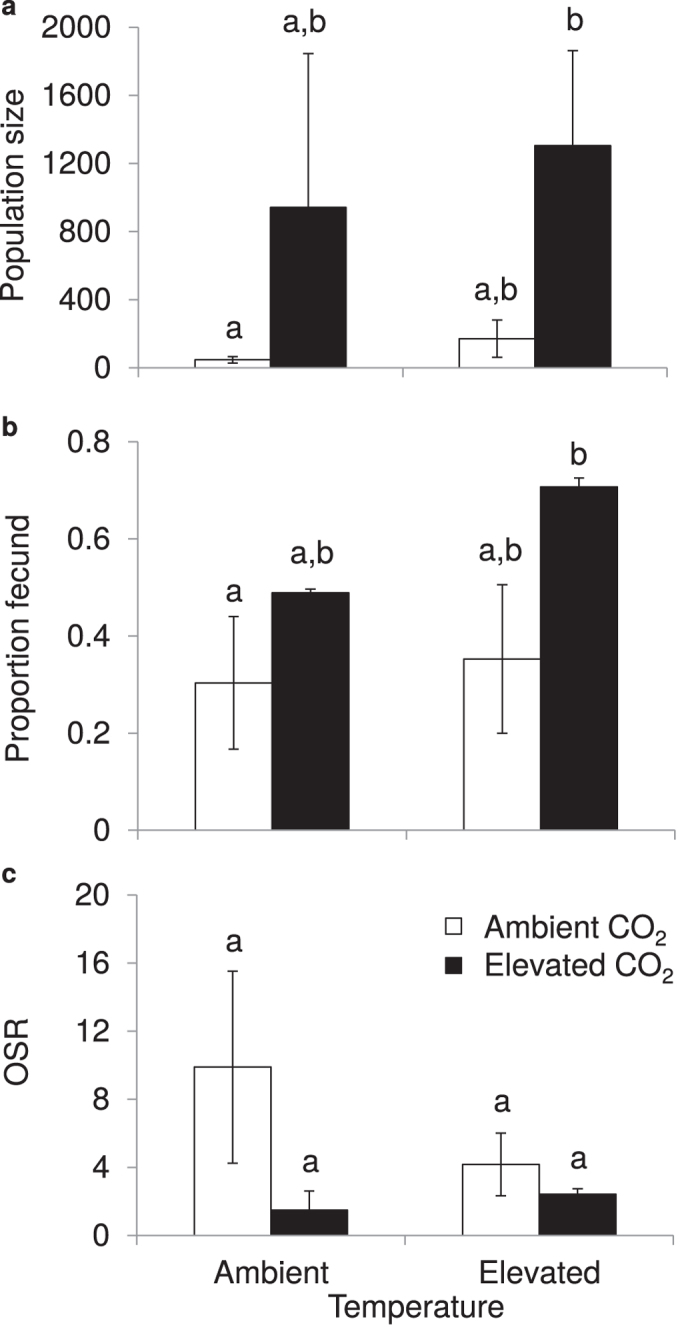
Effect of elevated temperature, elevated CO_2_, and the combination of elevated temperature and CO_2_ on population size and reproduction. Ambient and elevated temperature is on the x-axis with white bars representing ambient CO_2_ and black bars representing elevated CO_2_. (**a**) Average population size (±SEM; *n* = 12). (**b**) Average proportion of fecund females (±SEM; *n* = 12). Females with offspring within the brood pouch were considered fecund. (**c**) Average operational sex ratio (OSR, the ratio of fecund females to males) (±SEM; *n* = 12). Different letters represent statistically different (*P* < 0.05) means.

Populations exhibited changes after 3 months with increases in size occurring under the combination of elevated temperature and CO_2_ (i.e. future climate). Population sizes under future climate increased by at least 2500% relative to contemporary climate (Fig. 1a; Table S1; Χ^2^_3,7_ = 8.22, *P* = 0.042). The proportion of fecund females increased under future climate (Fig. 1b; Table S1, F_3,7_ = 4.63, *P* = 0.044), and there were fewer fecund females per male under future climate (Fig. 1c). However, the OSR did not differ between current and future climate (Table S1; F_3,6_ = 1.48, *P* = 0.31). Size frequency distributions in current and future climate had unimodal distributions at the end of the experiment (Fig. S1), reflecting overlapping generations driving population growth^35^.

**Figure 2:**
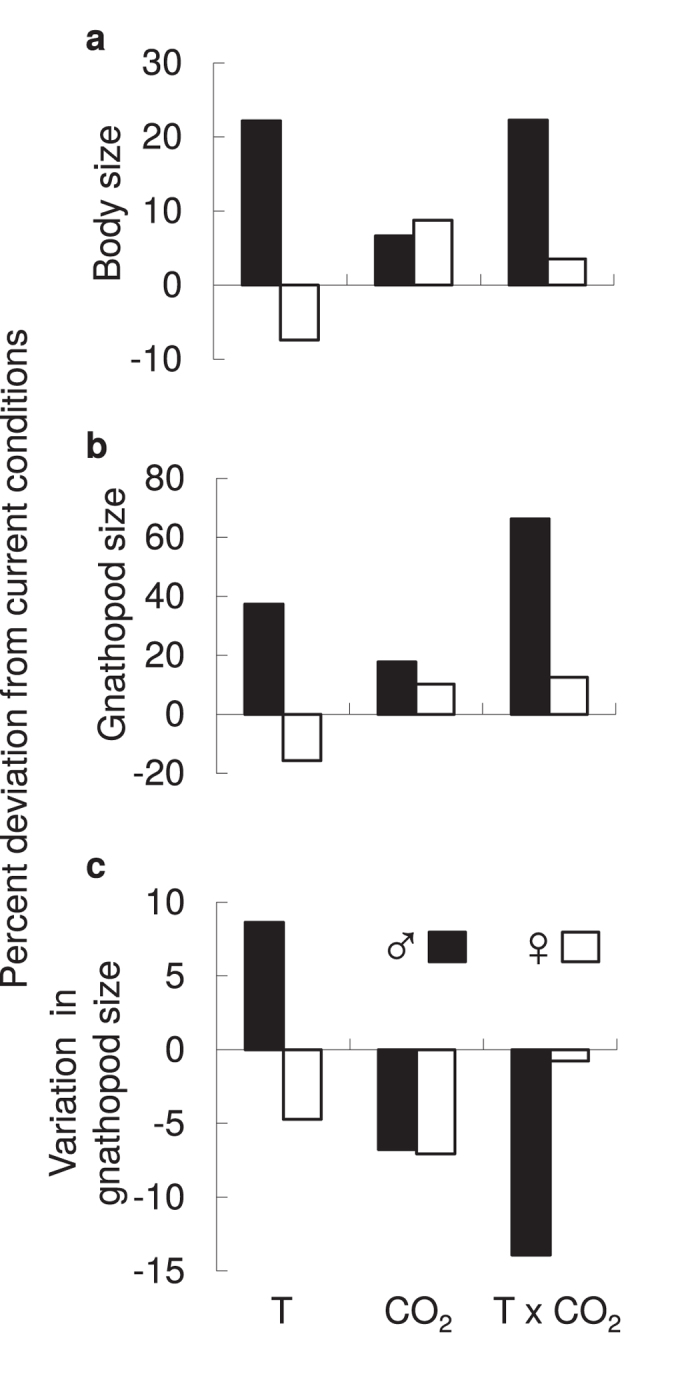
Selection on male and female traits under elevated temperature (T), carbon dioxide (CO_2_), and a combination of elevated temperature and carbon dioxide (T × CO_2_). Percent deviation from current conditions (control treatment) of (**a**) body size, (**b**) gnathopod size, and (**c**) variation in gnathopod size for males (black bars; *n* = 647) and females (white bars; *n* = 415). Deviation represents the treatment effect size (T, CO_2_ and T × CO_2_) relative to ambient; positive values represent an increase and negative values represent a decrease in average traits. Variation in gnathopod size is obtained by averaging the absolute value of studentized residuals from the gnathopod to body size regression.

Males and females responded differently to elevated temperature and CO_2_ (Fig. 2). Male amphipods were larger under future climate relative to current conditions (Fig. 2a), driven by the effect of elevated temperature (Fig. S2, Table S2; F_3,643_ = 23.24, *P* < 0.0001). In contrast, female body size did not differ among treatments (Fig. 2a; Fig. S2; Table S2; F_3,411_ = 1.22, *P* = 0.30). Male gnathopods dramatically increased in size under future climate compared to female gnathopods (Fig. 2b). Increases in male gnathopod size (Fig. S2; Table S3; F_7,639_ = 180.60, *P* < 0.0001) was driven by a synergistic effect of temperature and CO_2_ (Table S3; Current temperature: F_3,204_ = 119.99, *P* < 0.0001 and Elevated temperature: F_3,435_ = 241.52, *P* < 0.0001). Female gnathopod size decreased under elevated temperatures (Fig. S2; Table S3; F_7,407_ = 25.29, *P* < 0.0001). At a population level, male gnathopod variance was reduced (Fig. 2c) primarily due to elevated CO_2_ (Table S4; F_3,643_ = 3.08, *P* = 0.03) while variance in female gnathopod size (Fig. 2c) did not differ among treatments (Table S3; F_3,408_ = 0.41, *P* = 0.74).

The number of eggs produced (Table S5) did not differ among the treatments in our study. Egg number was a function of female body size (Table S5; F_7,232_ = 8.72, *P* < 0.0001), but female size did not consistently predict egg number across treatments (Table 1). In ambient conditions, egg number was independent of female size (Table 1), while temperature caused a disproportionate effect of female size on egg number. In contrast, elevated CO_2_ caused a linear increase in egg number with female size. When both elevated temperature and CO_2_ were present, female size had a significant non-linear effect on eggs produced (Table 1). Females experiencing elevated temperatures exponentially increased egg production, optimizing reproductive output (Table 1; Fig. S3) rather than increasing body size (Fig. 2a).

## Discussion

Treatments that created future climate conditions (i.e. the combination of elevated temperature and CO_2_) not only enhanced fecundity, but the apparent increase in reproductive success translated into greater population size over successive generations (Fig. S4). Increases in male body and gnathopod size were associated with an increase in the proportion of fecund females. As predicted from our model of sexual selection acting to buffer population growth, we observed wide variance in male gnathopod size in contemporary conditions, suggesting intense competition among individuals^19^,^36^, and a narrowing of this variance by future climate. Such reductions in variance of sexually selected traits reduce the intensity of male-male competition that is associated with decreased mate guarding and increased mating opportunities^18^,^36^. In addition, females shifted resource investment to egg production under elevated temperatures rather than enhancing size; life-history strategies were altered to optimize the number of eggs brooded under future climate, increasing population size. The relaxation of constraints on reproductive output was associated with enhanced food productivity in the same experiment (i.e. filamentous turfs^37^) and the associated reduction in predation^15^ could only have assisted in translating reproductive success into population growth over successive generations.

To assess whether future climate may boost intergenerational population growth, we need to understand how reproductive potential translates enhanced productivity to the next generation. Energy flows from producer to consumer will depend on both metabolic effects on the consumer^38^,^39^ and effects on the abundance and food quality of the producer recognizing variation among producer species^40^,^41^. For populations to grow, reproductive success needs to contribute and build upon both enhanced survivorship (i.e. reduced predation^15^) and individual growth; of which the latter is underpinned by greater metabolic demand^6^,^42^,^43^ and met by greater foraging activity^13^ of elevated primary productivity^39^. Population explosion under future climate is likely to be underpinned by the positive influence of elevated temperature on metabolic rate^7^, where requirements for greater food are met by the effect of carbon enrichment on algal resources^43^. Algal productivity can be supercharged by elevated temperature and carbon dioxide, particularly on fine, filamentous algae that herbivorous amphipods consume, but under current climate are normally ephemeral and sparse^10^. While there is recognition that temperature and carbon dioxide can ramp up primary productivity^44^, there has been less recognition of how this primary productivity may translate into an increase in secondary productivity. The lack of research that makes these connections between trophic levels has left some serious gaps in understanding for the stability of future food webs^12^. Here, we consider a critical mechanism that would allow for flexibility in mating strategy so individuals can adjust their reproductive investment^32^ when the concomitant increase in metabolic demand and population size is not constrained by resources or predators.

Future climate provides conditions that relax the drivers of variance in male traits. Strong selection on male sexually selected traits such as the amphipod’s gnathopod is affected by per capita resource availability^45^, such that reduced availability causes stress that widens variance in gnathopod size^32^. Widened variation among male gnathopod size within a population leads to disproportional mate guarding and competition, which has been shown to affect reproductive success in related species^36^. Female choice often selects for male sexually-selected traits^46^, and when females are less choosy, male trait variance decreases^47^. When females are less choosy, a higher proportion of females could become gravid^48^. Females are also likely to reach maturation more quickly under future climate, further boosting the effect of reduced choosiness on reproductive output. These results provide insight into short-term studies that demonstrate the ability of amphipods to quickly adapt to elevated CO_2_^49^ and temperature^50^, and naturally occur in greater abundance at CO_2_ seeps^12^. However, future studies should tease apart the effect of variation in male secondary characters driving fecundity under future climate conditions.

In conclusion, we suggest that intergenerational increases in population size might be facilitated by adjustments in reproductive success to favourable environmental conditions. For some consumers, therefore, future climate may not only increase individual growth and survival, but this added production ought to carry over to subsequent generations through increased reproductive success. Such population growth is normally buffered by mechanisms that govern reproductive success and its translation through survival to successive generations, but for many species we argue that the indirect effects of climate via resource provisioning and trophic control may reduce this buffering capacity. While the effects of future climate as a stressor has dominated research effort to date, we are only beginning to realise that it may also favour reproduction and population growth of some species which can also boom under these same conditions.

## Methods

*Cymadusa pemptos* is a common Ampithoid herbivore in temperate kelp forests of South Australia with preferred feeding on benthic turfs (Heldt *unpub*.). Males have larger gnathopods than females and both sexes build and inhabit nests among subtidal algae, such as seagrass (0.6–0.8 m depth^51^) and kelp beds (3–5 m depth). *Cymadusa pemptos* reaches maturity in an estimated 4–6 weeks, and females produce between 10 and 19 eggs (Heldt *unpub*.), similar to other *Cymadusa* species where females can live for up to 4 weeks after releasing brood^52^,^53^,^54^. To test responses of herbivorous *C. pemptos* populations to future climate, we designed an experiment using mesocosms with a crossed design of elevated temperature and CO_2_. We refer to future climate conditions as the combined effects of elevated temperature and elevated CO_2_.

Flow-through mesocosm tanks (2,000 L, n = 12) were established at the South Australian Research and Development Institute, in West Beach, South Australia (34.9453 °S, 138.5038 °E) in September 2013. An incoming flow rate (4 L min^−1^) of filtered natural seawater (salinity ~40 ppt) was used to maintain water quality, and all mesocosms experienced natural spring and summer daylight cycles for South Australia. Temperature and CO_2_ were manipulated to reflect ambient conditions of the sites in which organisms were sourced (i.e., current temperature and atmospheric CO_2_ concentrations^44^), and treatments of future climate were set using predictions in the 2007 IPCC report^55^. Each mesocosm was maintained as an independent replicate. Within each replicate mesocosm, individual heater/chiller units and independent adjustments to CO_2_ input maintained target temperature and CO_2_ conditions. Heater/chiller units (TC-60 Aquarium Chillers, TECO Refrigeration Technologies, Ravenna, Italy), CO_2_ generated by gas mixers (Pegas 4000 MF, Columbus Instruments, Columbus Ohio USA), and water flow were independently manipulated for each mesocosm^56^.

Each mesocosm was stocked with a local community of primary producers (five kelp holdfasts and fronds at 1.2 kg [*Ecklonia radiata*] and five seeded fibre-board tiles (10 cm × 10 cm) containing a mixed assemblage of filamentous algae^37^), herbivores (five sea urchins [*Heliocidaris erythrogramma*], fifteen marine gastropods [*Turbo undulatus*], and *Cymadusa pemptos* amphipods). Predators were also present, one crab (*Ozius truncatus*), three sharks (*Heterodontus portusjacksoni*^15^, and one spiny lobster (*Jasus edwardsii*). To ensure that experimental densities reflected natural densities, we seeded mesocosms with kelp habitat harboring natural *C. pemptos* populations. To collect amphipods with kelp, each kelp was entirely enclosed with a plastic bag in the field with the holdfast removed intact, thus sealing in natural amphipod densities^57^,^58^,^59^. Amphipod abundances were homogenized across tanks as all kelp fronds were kept in holding tanks prior to placement in each of the mesocosms with an estimated 100 amphipods and OSR of 6:1. The multi-trophic level mesocosms lasted multiple generations of amphipods. Use of vertebrates in these experiments, described in previously published work^15^, were approved by the University of Adelaide animal ethics committee (permit: S-2013-095) and in accordance to the University’s animal ethics guidelines. Shark collections were carried out with permission of the South Australian Government Department of Primary Industry and Regions SA (permit: 990295).

Temperature and pH were measured at different times of the day every day for each tank^56^; this sampling revealed that noon measurements were representative of the environmental conditions of the tank for that day. Total alkalinity (T_A_) was measured weekly for each tank and used for pCO_2_ calculations. Temperature and pH differed between ambient and elevated conditions and followed natural daily fluctuations throughout the entire experiment^56^. The average temperature and pH for current conditions (C; ambient temperature and CO_2_) were 15.4 °C ± 0.1 SE and 8.16 ± 0.01 SE respectively. In contrast, future conditions (T × CO_2_; elevated temperature and CO_2_) had a temperature of 17.9 °C ± 0.3 SE and pH 7.99 ± <0.01 SE. The elevated temperature treatment (T, elevated temperature and ambient CO_2_), had a temperature of 18.1 °C ± 0.2 SE and pH 8.15 ± 0.01 SE. Finally, in the elevated CO_2_ treatment (CO_2_; ambient temperature and elevated CO_2_) the average temperature was 15.4 °C ± 0.1 SE and the average pH was 8.01 ± 0.01 SE. For all treatments, pH was calculated from back-transformed hydrogen ion concentrations. Total alkalinity was measured using a potentiometric titrator (888 Titrando, Metrohom, USA) and pCO_2_ was determined using CO_2_SYS program for Excel^56^,^60^. Average alkalinity and pCO_2_ measures differed among C (T_A_ = 2563 μmol kg^−1^ ± 3, *p*CO_2_ = 381 μatm ± 38), T (T_A_ = 2562 μmol kg^−1^ ± 10, *p*CO_2_ = 427 μatm ± 42), CO_2_ (T_A_ = 2555 μmol kg^−1^ ± 5, *p*CO_2_ = 607 μatm ± 52) and T × CO_2_ (T_A_ = 2547 μmol kg^−1^ ± 2, *p*CO_2_ = 663 μatm ± 80) treatments.

In December 2013, 13 weeks after stocking mesocosms, kelp fronds from each mesocosm were removed, dipped into freshwater, and gently wiped; this allowed us to collect amphipods that had undergone 2–3 generations in experimental conditions. The freshwater containing amphipods was filtered through a 0.5 mm mesh and amphipods were preserved in 100% ethanol. All adults were accounted for and photographed; juveniles were subsampled by stirring and removing 10% of the solution and collected individuals were separated for photographic analysis. Photographs were taken using a Canon G10 camera fitted with a high definition 10x macro lens, and body size (mm length) for all individuals and gnathopod size (i.e. the second appendage, mm length from the tip of the dactyl to the base of the propod when the chelae were closed) for adults were measured using ImageJ^61^.

We first analysed population-level responses to elevated temperature and elevated CO_2_ treatments. Amphipod population size was compared among treatments using a generalized linear model with a normal error distribution and two levels of CO_2_ and temperature treatments (i.e. ambient and elevated). The proportion of fecund females and operational sex ratio (OSR) were compared among treatments using two-way ANOVAs. The proportion of fecund females was arcsin transformed, and OSR was calculated as the number of fecund females relative to the number of males in a given population and log transformed.

Next, we analysed individual-level responses to the experimental treatments. Body size, gnathopod size, and the number of eggs per female were log transformed. Changes in body size between treatments were tested for each sex using a two-way ANOVA. Differences in gnathopod size were compared using a two-way ANOVA with body size as a covariate. Significant 3-way interactions between the two treatments and the covariate were further analysed for each level of temperature treatment (i.e. current and elevated) using an ANCOVA with CO_2_ as a fixed effect and body size as a covariate. To explore population-level variation in gnathopod size between treatments, we obtained studentized residuals from a gnathopod to body size regression of each treatment and sex and used the absolute value of studentized residuals to calculate mean residual variation (i.e., reflecting variation in gnathopod size independent of body size). Variation in gnathopod size was square root transformed, and a two-way ANOVA tested for treatment effects. To represent changes under future climate relative to current conditions, we calculated the difference between future and current condition means (e.g., either body size, gnathopod size and gnathopod variation) standardized by the current mean. Finally, the number of eggs produced by females was compared across treatments with a two-way ANOVA using female body size as a covariate. Relationships between body size and egg production were non-linear, and to further analyse relationships, we regressed the number of eggs per female against female body size for each of the treatment using second-order polynomial regressions with log-transformed data. We ensured data met parametric assumptions and set alpha = 0.05; analyses were carried out in JMP (SAS Institute, Cary, NC).

## Additional Information

**How to cite this article**: Heldt, K. A. *et al.* Future climate stimulates population out-breaks by relaxing constraints on reproduction. *Sci. Rep.* **6**, 33383; doi: 10.1038/srep33383 (2016).

## Supplementary Material

Supplementary Information

## Figures and Tables

**Table 1:** Polynomial regressions of the number of eggs produced per female against female body size for each of the four treatment conditions.

Treatment	Intercept	Body (x)	Body^2^ (x^2^)
Ambient	0.318 (0.933)	0.745 (0.667)	5.554 (0.623)
Elevated Temperature	−0.942 (0.394)	**1.428 (0.006)**	**3.043 (0.036)**
Elevated CO_2_	**−2.420 (0.017)**	**2.131 (<0.0001)**	0.931 (0.449)
CO_2_ × Temperature	**−1.512 (0.022)**	**1.638 (<0.0001)**	**3.019 (0.009)**

Data were log-transformed before analysis. Numbers in parentheses are the P-values testing for similarities against an intercept=0 or slope=0.
