# Supplementary material for: Future climate stimulates population out-breaks by relaxing constraints on reproduction
Source: Sci Rep. 2016 Sep 14;6:33383. doi: 10.1038/srep33383 (PMC5022049; doi:10.1038/srep33383)
Supplement: Supplementary Information [file srep33383-s1.pdf]

Title: Future climate stimulates population out-breaks by relaxing constraints on reproduction

Authors: Katherine A. Heldt, Sean D. Connell, Kathryn Anderson, Bayden D. Russell, Pablo Munguia

Supplementary Information

Figure S1, S2, S3, S4

Tables S1, S2, S3, S4, S5

Figure S1

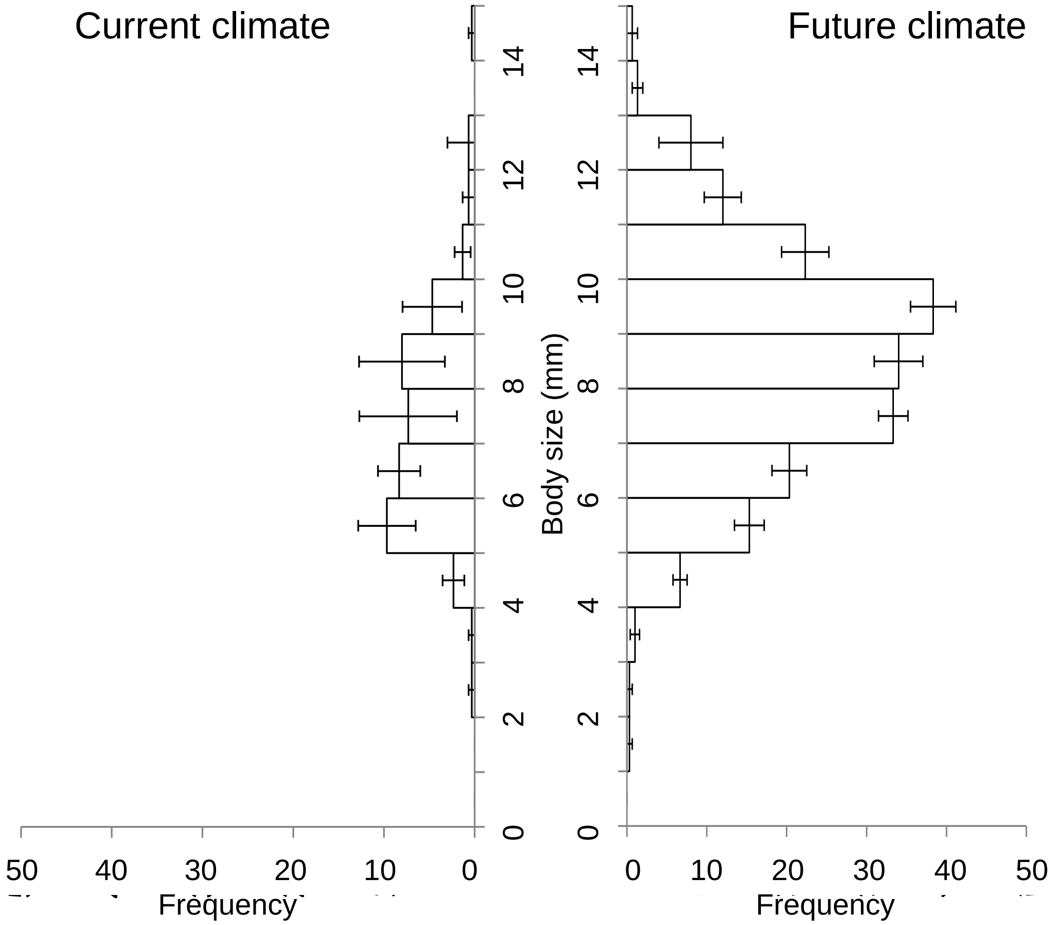

Figure S1 Body size (mm length) frequency distributions of *C. pemptos* populations under current (left panel) and future climate conditions (right panel) (Means ± SEM for each size class). Average body size in current climate conditions was 7.5 ± 0.3 and in future climate conditions was 8.5 ± 0.1.

Figure S2

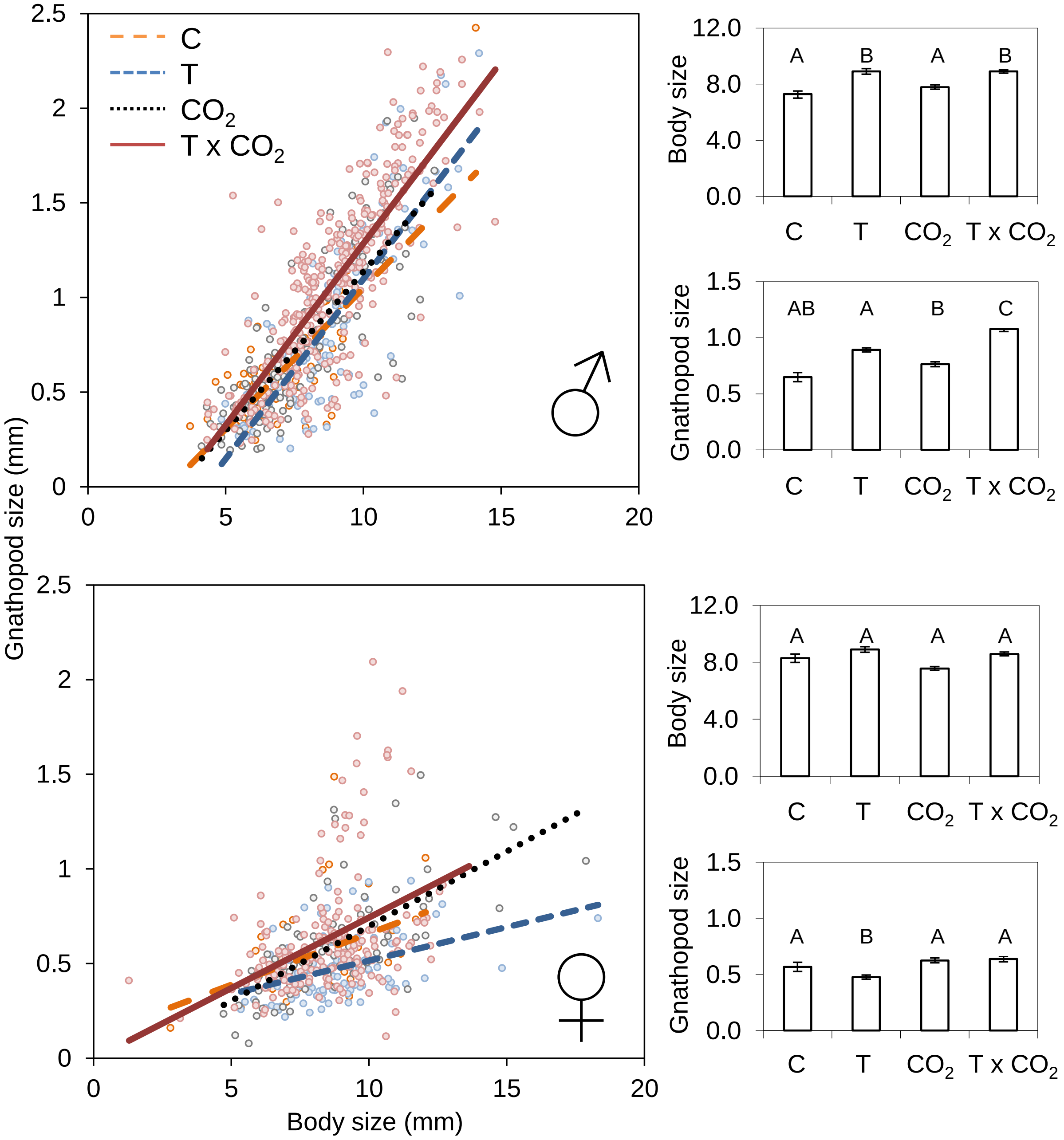

Figure S2 Gnathopod size to body size (mm length) regressions and average body and gnathopod sizes under ambient temperature and CO_2 (C; orange line, long dashes), elevated temperature (T; blue line, short dashes), elevated CO_2 (CO_2; black line, dotted), and elevated temperature and CO_2 (T x CO_2; red line, solid) conditions. Different letters represent statistically different ($P < 0.05$) means. ***Top*** Males, ***bottom*** Females.

Figure S3

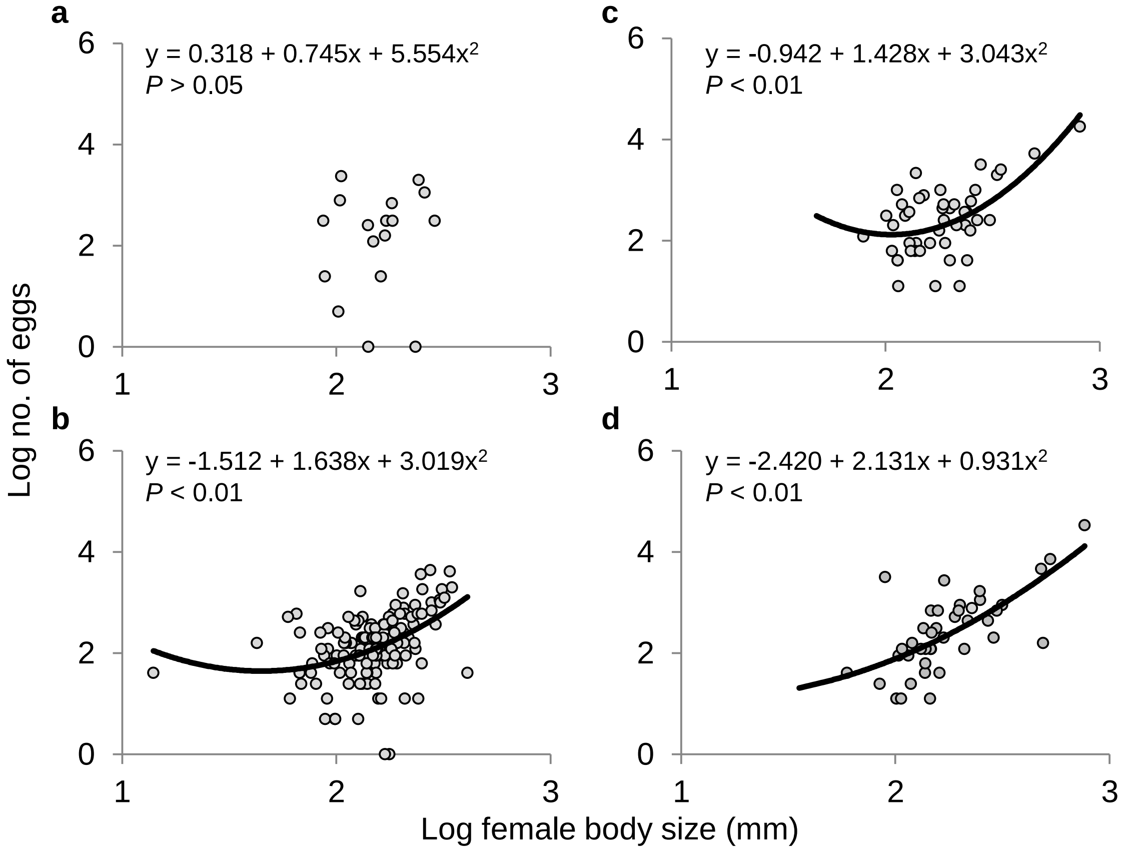

Figure S3 Polynomial regressions of the number of eggs produced per female to female body size (mm length) under a) current climate (ambient temperature and ambient CO_2), b) future climate (elevated temperature and elevated CO_2), c) elevated temperature and ambient CO_2, and d) ambient temperature and elevated CO_2. Note the log scale on the x and y-axis.

Figure S4

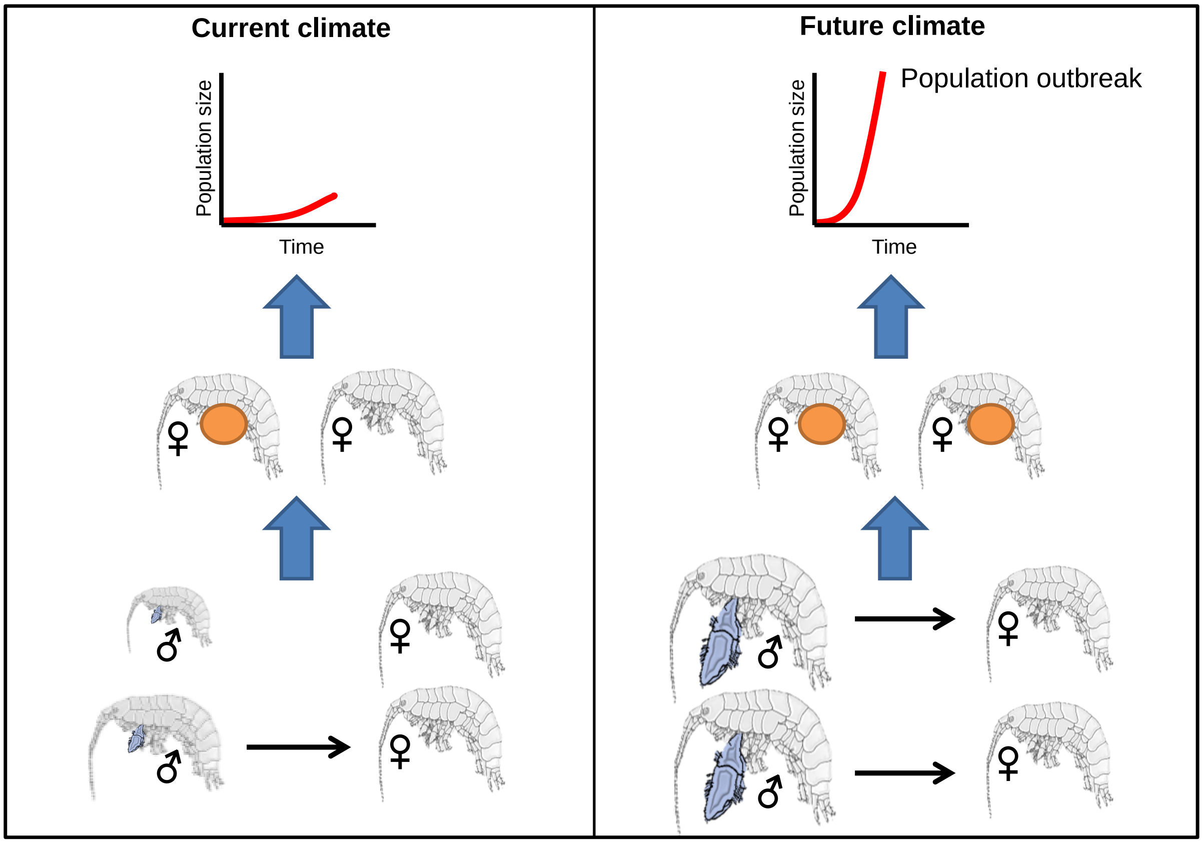

Figure S4 Population outbreaks of *Cymadusa pemptos* under current and future climates. Relative to current climate, the proportion of gravid females doubled and variance in phenotypic variation of male secondary sexual characters (i.e. gnathopods; highlighted in blue) was significantly reduced under future climate. Black arrows represent mating success with fecund females brooding eggs (orange circles). Amphipod image modified from Peart 2007.

Tables S1-S4

Table S1. Results from a generalized linear model testing the effect of CO_2 and temperature on population size, and a two-way ANOVA testing these treatments on the proportion of fecund females and operational sex ratio (OSR).

	Population Size			Proportion Fecund			OSR		
**Full Model**	**df**	**X^2**	**p-value***	**df**	**F-value**	**p-value***	**df**	**F-value**	**p-value***
	3,7	8.22	**0.0415**	3, 7	4.63	**0.0435**	3, 6	1.48	0.3118
**Main Effects**		X^2	p-value*		F-value	p-value*		F-value	p-value*
CO_2 (C)		**5.19**	**0.0228**		2.55	0.1543		0.07	0.7977
Temp (T)		0.81	0.3680		**9.15**	**0.0192**		3.19	0.1245
C x T		0.03	0.8669		1.24	0.3025		0.85	0.3932
*significant effects and interactions, *P*<0.05, are in bold									

Table S2. Two-way ANOVAs testing the effect of CO_2 and temperature on male and female body size (mm length).

	Male Body Size			Female Body Size		
**Full Model**	**df**	**F-value**	**p-value***	**df**	**F-value**	**p-value***
	3, 643	23.14	**<0.0001**	3, 411	1.22	0.30
**Main Effects**		F-value	p-value*		F-value	p-value*
CO_2 (C)		1.95	0.1631		0.06	0.8117
Temp (T)		62.49	**<0.0001**		2.29	0.1313
C x T		1.74	0.1871		1.69	0.1937

*significant effects and interactions, P<0.05, are in bold

Table S3. Two-way ANOVAs testing the effect of CO_2 and temperature on male and female gnathopod size (mm length) using body size as a covariate. Because of a significant 3-way interaction, we then performed an ANCOVA for each temperature treatment testing differences between CO_2 treatments with body size as a covariate. P-values <0.05 are in bold.

	Male Gnathopod Size			Female Gnathopod Size		
**Full Model**	**df**	**F-value**	**p-value***	**df**	**F-value**	**p-value***
	7, 639	180.60	**<0.0001**	7, 407	25.29	**<0.0001**
**Main Effects**		F-value	p-value*		F-value	p-value*
CO_2 (C)		26.04	**<0.0001**		10.21	**0.0015**
Temp (T)		3.02	0.0829		2.54	0.1116
C x T		5.04	**0.0251**		11.97	**0.0007**
Log Body (B)		697.80	**<0.0001**		122.70	**<0.0001**
C x B		1.85	0.1742		1.93	0.3343
T x B		4.63	**0.0319**		2.10	**0.0215**
C x T x B		7.83	**0.0053**		0.61	0.0966

ANCOVAs for each temperature treatment.

**Ambient temperature**	Male Gnathopod Size			Female Gnathopod Size		
**Full Model**	**df**	**F-value**	**p-value***	**df**	**F-value**	**p-value***
	3, 204	119.99	**<0.0001**	3,122	34.38	**<0.0001**
**Main Effects**		F-value	p-value*		F-value	p-value*
CO_2 (C)		0.59	0.4426		0.08	0.78
Log Body (B)		242.94	**<0.0001**		70.77	**<0.0001**
C x B		7.13	**0.0082**		3.09	0.08
**Elevated temperature**	Male Gnathopod Size			Female Gnathopod Size		
**Full Model**	**df**	**F-value**	**p-value***	**df**	**F-value**	**p-value***
	3, 435	241.52	**<0.0001**	3,285	26.96	**<0.0001**
**Main Effects**		F-value	p-value*		F-value	p-value*
CO_2 (C)		42.45	**<0.0001**		35.45	**<0.0001**
Log Body (B)		544.50	**<0.0001**		40.69	**<0.0001**
C x B		1.38	0.2410		0.31	0.58

*significant effects and interactions, P<0.05, are in bold

Table S4. Two-way ANOVAs testing the effect of CO2 and temperature on variance in male and female gnathopod size (mm length).

	Male Gnathopod Variance			Female Gnathopod Variance		
**Full Model**	**df**	**F-value**	**p-value***	**df**	**F-value**	**p-value***
	3, 643	3.08	**0.0269**	3, 408	0.41	0.7447
**Main Effects**		F-value	p-value*		F-value	p-value*
CO_2 (C)		26.04	**0.0225**		0.97	0.3262
Temp (T)		3.02	0.2467		0.07	0.7873
C x T		5.04	0.8246		0.24	0.6268

*significant effects and interactions, *P*<0.05, are in bold

Table S5. Two-way ANOVA testing the effect of CO_2 and temperature on the number of eggs per female using body size as a covariate.

	Number of Eggs		
**Full Model**	**df**	**F-value**	**p-value***
	7, 232	8.72	**<0.0001**
**Main Effects**		F-value	p-value*
CO_2 (C)		0.24	0.6222
Temp (T)		0.06	0.8035
C x T		1.71	0.1925
Log Body (B)		**21.23**	**<0.0001**
C x B		0.08	0.7771
T x B		0.09	0.7588
C x T x B		**5.08**	**0.0251**

*significant effects and interactions, P<0.05, are in bold
